# Supplementary material for: Influence of Dispersion and Orientation on Polyamide-6 Cellulose Nanocomposites Manufactured through Liquid-Assisted Extrusion
Source: Nanomaterials (Basel). 2022 Feb 28;12(5):818. doi: 10.3390/nano12050818 (PMC8912402; doi:10.3390/nano12050818)
Supplement: Supplementary file 1 [file nanomaterials-12-00818-s001.zip › nanomaterials-1611262-supplementary.pdf]

nanomaterials

Supplementary Materials

Influence of Dispersion and Orientation on Polyamide-6 Cellulose Nanocomposites Manufactured through Liquid-Assisted Extrusion

Luísa Rosenstock Völtz [1,2], Shiyu Geng [1,2], Anita Teleman [3] and Kristiina Oksman [1,2,4,*]

1 Division of Materials Science, Department of Engineering Sciences and Mathematics, Luleå University of Technology, SE-97187 Luleå, Sweden; luisa.voltz@ltu.se (L.R.V.); shiyu.geng@ltu.se (S.G.)
2 Wallenberg Wood Science Center (WWSC), Luleå University of Technology, SE-97187 Luleå, Sweden
3 RISE Research Institutes of Sweden, SE-11486 Stockholm, Sweden; anita.teleman@ri.se
4 Department of Mechanical & Industrial Engineering (MIE), University of Toronto, Toronto, ON M5S 3G8, Canada
* Correspondence: kristiina.oksman@ltu.se

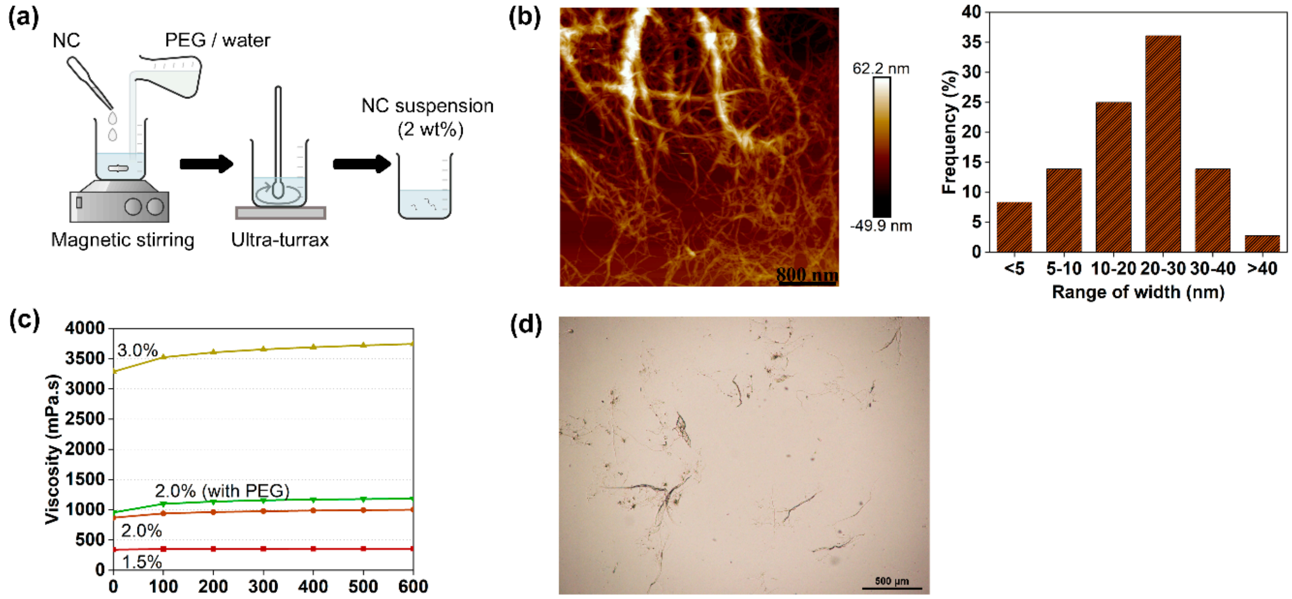

Figure S1. (**a**) NC suspension preparation by adding water and/or PEG following by magnetic stirring and ultra turrax; (**b**) AFM micrograph of the nanocellulose (0.01 wt.%) and width distribution of the nanocellulose; (**c**) viscosity versus time curves for different NC concentrations (1.5, 2 and 3 wt.% water base and 2 wt.% PEG/water base) and (**d**) OM image of NC suspension (0.01 wt.%), scale bar 500 µm

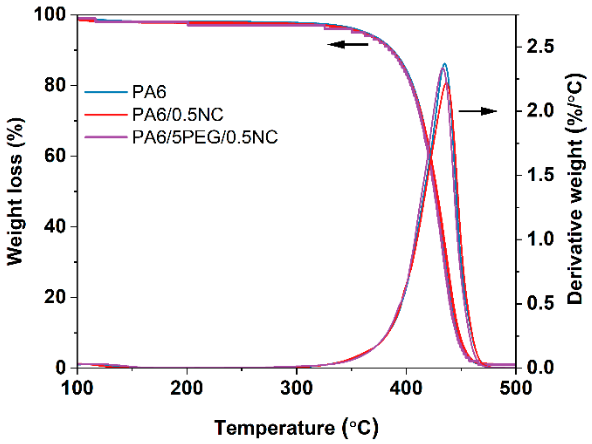

Figure S2. Thermogravimetric curves for PA6 and PA6-nanocomposites measured by TGA. The onset degradation temperature for PA6: 403 °C, PA6/0.5NC: 402 °C, and PA6/5PEG/0.5NC: 402 °C

Table S1. Degree of crystallinity (X_c) and melting peaks measured by DSC. The samples were dried prior testing.

Sample	X_c (%)	T_{m1} (°C)	T_{m2} (°C)
PA6	21	205	218
PA6/0.5NC	28	200	218
PA6/5PEG/0.5NC	25	205	218
OPA6	31	210	221
OPA6/0.5NC	42	212	222
OPA6/5PEG/0.5NC	30	210	221

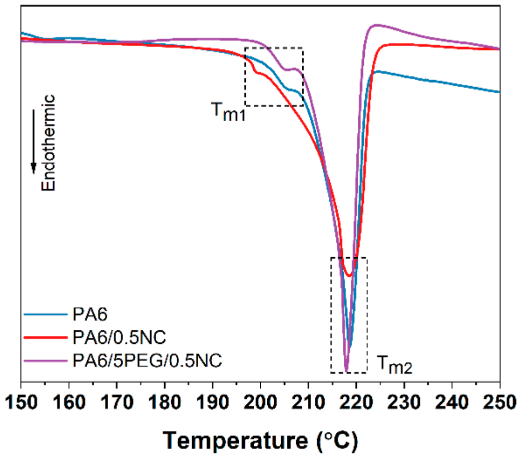

Figure S3. DSC curves for PA6 and PA6-nanocomposite. The double melting peaks, T_{m1} and T_{m2}, are shown in the graph

Table S2. Tensile properties of non-oriented PA6 and PA6-nanocomposites. The toughness was calculated by the area under the stress-strain curve

Sample	Strength (MPa)	Modulus (GPa)	Elongation at break (%)	Toughness (MJ/m³)
PA6	61 ± 2 [A]	2.0 ± 0.1 [A]	21 ± 8 [A]	11.5 ± 4.4 [A]
PA6/0.5NC	60 ± 1 [A]	2.3 ± 0.1 [B]	19 ± 6 [A]	10.4 ± 2.9 [A]
PA6/5PEG/0.5NC	52 ± 1 [B]	1.4 ± 0.1 [C]	63 ± 4 [B]	29.5 ± 2.5 [B]

[A,B,C] Marked with the same letter within the same column are not significantly different at a 5% significance level based on ANOVA and Tukey's Test.

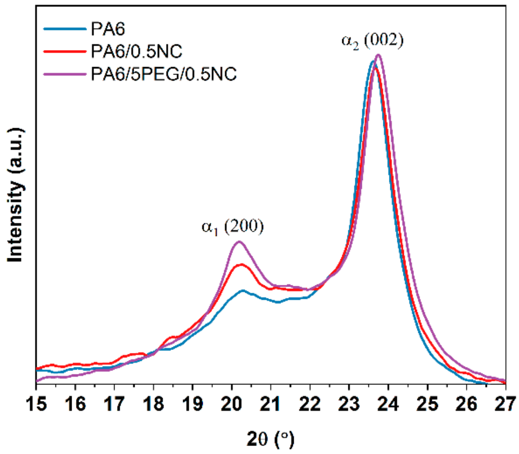

Figure S4. 1D-XRD for PA6 and PA6-nanocomposite. The first peak around 20.2° is related to α_1 crystals (200) and the second peak around 23.7° to α_2 crystals (002)

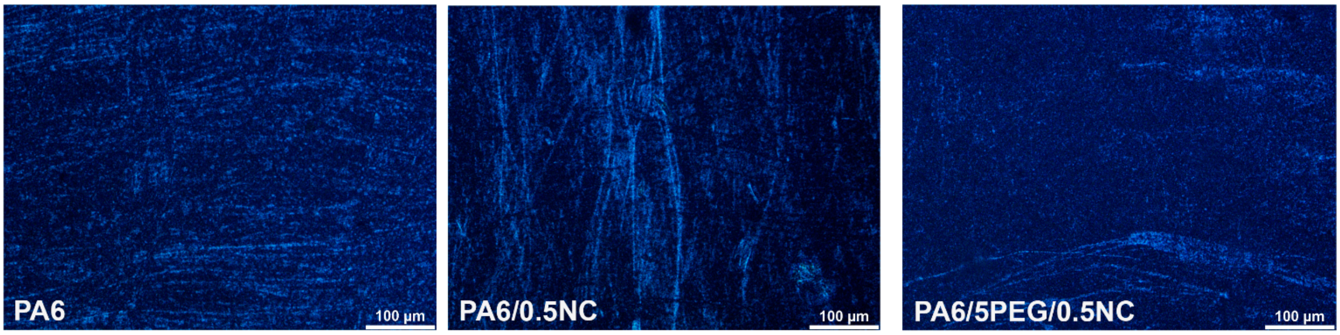

Figure S5. POM using hot-stage (220 °C) for PA6 and PA6-nanocomposites at 90 seconds (scale bar 100 µm).

Table S3. Tensile properties of oriented PA6 and PA6-nanocomposites. The toughness was calculated by the area under the stress-strain curve

Sample	Strength (MPa)	Modulus (GPa)	Elongation at break (%)	Toughness (MJ/m³)
OPA6	203 ± 5[A]	2.6± 0.1[A]	24± 4[A]	34.0±7.6[A]
OPA6/0.5NC	215 ± 7[B]	3.0± 0.2[B]	18± 3[B]	24.3±6.2[B]
OPA6/5PEG/0.5NC	221 ± 5[B]	2.8± 0.2[A/B]	23± 1[A]	33.6±3.4[A/B]

[A,B,C] Marked with the same letter within the same column are not significantly different at a 5% significance level based on ANOVA and Tukey's Test.

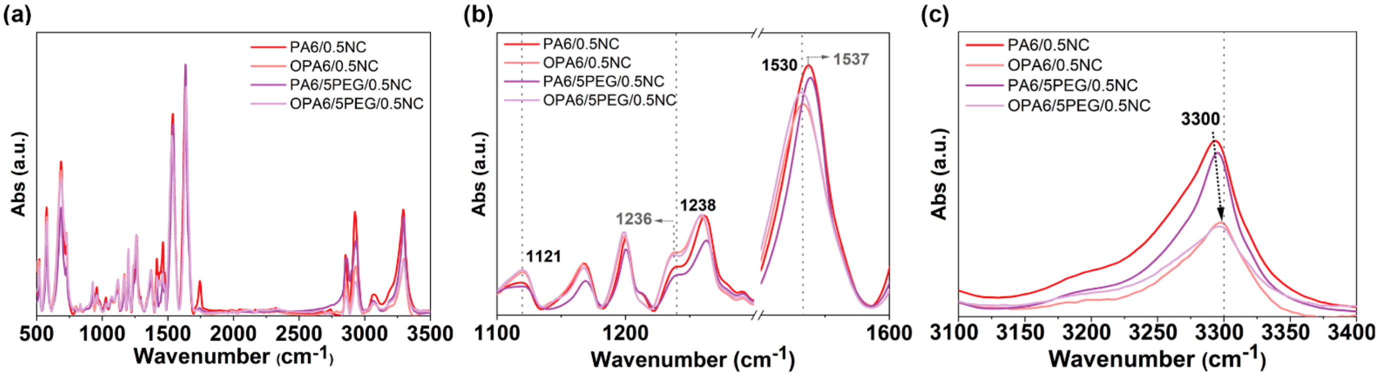

Figure S6. FT-IR spectra for PA6-nanocomposites and OPA6-nanocomposites, (**a**) FTIR spectra from 500 to 3500 cm^{-1}; (**b**) from 1100 to 1600 cm^{-1}; and (**c**) from 3100 to 3400 cm^{-1}.

Table S4. Apparent crystallite size and diffraction angles measured by 1D-XRD scattering patterns

Sample	Crystallite size (nm)	$2\theta_1$ (°)	$2\theta_2$ (°)
PA6	5.32	20.3	23.6
PA6/0.5NC	7.79	20.2	23.7
PA6/5PEG/0.5NC	6.70	20.2	23.8
OPA6	3.88	20.6	23.2
OPA6/0.5NC	4.50	20.5	23.1
OPA6/5PEG/0.5NC	2.64	20.5	23.3

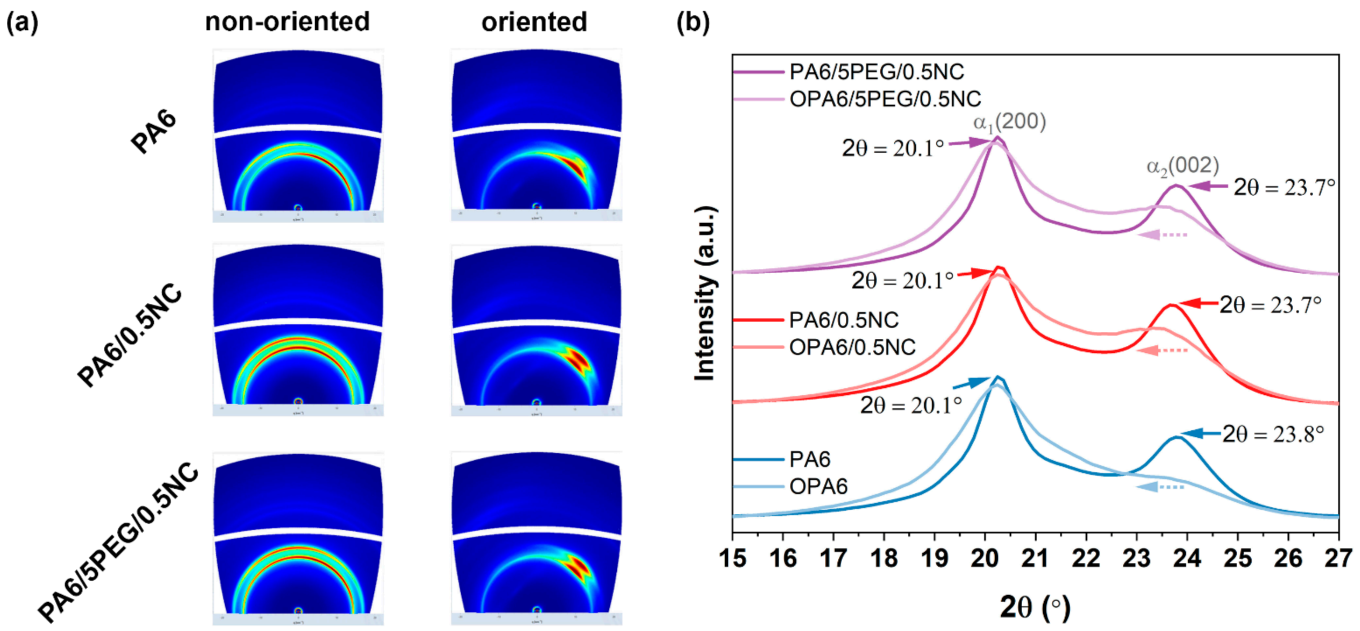

Figure S7. (**a**) 2D-WAXS diffractograms and (**b**) 2D-XRD scattering pattern for PA6 and PA6-nanocomposites before and after SSD

Table S5. Orientation index for crystalline PA6 in the composites, as estimated from WAXS measurements. If all crystalline PA6 is aligned in the same direction, f_c = 1, and if it is randomly distributed, f_c = 0

**Sample**	**Orientation index, f_c [1]**	
	Non-oriented	Oriented
PA6	0.60; 0.53	0.82; 0.83
PA6/0.5NC	0.32; 0.37	0.84; 0.84
PA6/5PEG/0.5NC	0.39; 0.42	0.86; 0.85

[1] Azimuthal integration of the γ(001)(200), α(200) and α(002) scattering planes, 2θ = 20.1 ± 0.4° ; 23.7 ± 0.4°.
